# Prevalence, diagnosis, and manifestations of brucellosis: A systematic review and meta-analysis

**DOI:** 10.3389/fvets.2022.976215

**Published:** 2022-12-22

**Authors:** Saeed Khoshnood, Reza Pakzad, Maryam Koupaei, Maryam Shirani, Almas Araghi, Golnaz Mokhtari Irani, Melika Moradi, Iraj Pakzad, Nourkhoda Sadeghifard, Mohsen Heidary

**Affiliations:** ^1^Clinical Microbiology Research Center, Ilam University of Medical Sciences, Ilam, Iran; ^2^Student Research Committee, Ilam University of Medical Sciences, Ilam, Iran; ^3^Department of Epidemiology, Faculty of Health, Ilam University Medical Sciences, Ilam, Iran; ^4^Department of Microbiology and Immunology, School of Medicine, Kashan University of Medical Sciences, Kashan, Iran; ^5^Toxicology Research Center, Medical Basic Sciences Research Institute, Ahvaz Jundishapur University of Medical Sciences, Ahvaz, Iran; ^6^Department of Microbiology, Faculty of Biological Sciences, North Tehran Islamic Azad University, Tehran, Iran; ^7^Department of Virology, School of Medicine, Ahvaz Jundishapur University of Medical Sciences, Ahvaz, Iran; ^8^Department of Microbiology, School of Medicine, Ahvaz Jundishapur University of Medical Sciences, Ahvaz, Iran; ^9^Department of Medical Microbiology, Faculty of Medicine, Ilam University of Medical Science, Ilam, Iran; ^10^Department of Laboratory Sciences, School of Paramedical Sciences, Sabzevar University of Medical Sciences, Sabzevar, Iran; ^11^Cellular and Molecular Research Center, Sabzevar University of Medical Sciences, Sabzevar, Iran

**Keywords:** brucellosis, diagnosis, prevalence, *Brucella*, review

## Abstract

**Objectives:**

Brucellosis is one of the most prevalent zoonotic diseases common between humans and animals. Despite eradication efforts, the burden of the disease is well-known in endemic countries and in countries where brucellosis has not been an important health issue until recently. The aim of this study was to evaluate the prevalence, diagnosis, and manifestations of brucellosis.

**Methods:**

In this study, PubMed, Web of Science, Scopus, Embase, and Google scholar databases were systematically searched to find studies published from 2011 to 2021. The search was conducted using text words and Medical Subject Headings (MeSH) Terms on the prevalence of brucellosis. Stata software 14.0 was used for all analyses.

**Results:**

Based on the results, the pooled prevalence of brucellosis was 15.27% (95% CI: 9.68–21.86; heterogeneity *I*^2^ index: 97.43; *p* < 0.001) for man and 15.33% (95% CI: 7.19–25.75; heterogeneity *I*^2^ index: 98.19; *p* < 0.001) for woman. Age (coefficient: 0.240; *p* = 0.480), gender (coefficient: −0.017; *p* = 0.800), and publication year (coefficient: 0.114; *p* = 0.861) showed no significant effect on heterogeneity among studies. Egger's test indicated a significant publication bias for the prevalence of brucellosis (coefficient 3.894; *p* < 0.001). Moreover, the trim-and-fill method exhibited that the adjusted prevalence of brucellosis (18.30%, 95% CI: 14.10–22.52) was not significantly different from the original prevalence of brucellosis.

**Conclusion:**

The pooled estimate for brucellosis prevalence was estimated as 15.53%. To better understand the epidemiology of brucellosis globally, more extensive studies are needed to be conducted throughout the world, especially in developing and low-income countries.

## Introduction

Brucellosis is one the most common zoonotic diseases affecting 500,000 cases annually (1). This disease was formerly known by names such as Malta fever, Mediterranean fever, Gibraltar fever, Cyprus fever, and Undulant fever (2). Among several species of *Brucella* identified*, B. melitensis, B. abortus*, and *B. suis* are the most and *B. canis* is the least important causative factor of human diseases (1, 2). To date, no disease has been reported to be caused by *B. ovis* and *B. neotomae* (1). Brucellosis has a wide range of clinical manifestations that frequently lasts from a few days to several years (3). In humans, the disease is rarely fatal but generally debilitating (4). It has various routes of transmission, including the gastrointestinal tract, respiratory tract, skin, and mucous membranes, as well as contact with body fluids. Disease transmission from animal to human occurs mainly through the consumption of raw meat and dairy products. Brucellosis is often misdiagnosed, which, in turn, leads to prolonged illness and inadequate treatment (3). Moreover, the symptoms of the disease are not specific and make it difficult to diagnose. The prevailing epidemiological situation of brucellosis in susceptible animals (livestock and wildlife) in a country or region plays a significant role in choosing a particular diagnostic test strategy. Diagnostic tests can be used for a variety of purposes, including confirmatory diagnosis, screening or prevalence studies, and confirmation of disease. In countries where brucellosis is being eradicated, surveillance is needed to prevent the reintroduction of brucellosis through importing infected animals or animal products. The validity of such diagnostic tests, especially in wildlife, is still an issue (5).

Diagnosis of brucellosis is conducted directly (bacteriological and molecular methods) and indirectly (*in vitro* serological methods and allergic methods *in vivo*). The “gold standard” for diagnosing this disease is direct bacteriological testing, that is, cultivation of *Brucella* isolated from body fluids or tissues. However, to circumvent the problems of bacteriological testing, molecular biological techniques, which are often based on polymerase chain reaction (PCR) amplification, are promisingly utilized to identify different types of *Brucella* species (6). Serological tests are very important in diagnosis but are often difficult to interpret (7). These methods are used for the initial screening of human brucellosis and also during the subsequent treatment. Serial serological tests are commonly recommended because of the frequent false-negative serological tests in the early days of infection and due to their capability of correct diagnosis and monitoring for response to therapy (8). Rose Bengal test (RBT), real-time PCR, serum agglutination test (SAT), and complement fixation test (CFT) are diagnostic methods for brucellosis (9). However, none of these tests can alone diagnose all different stages of brucellosis. Therefore, combined tests can be applied for definitive diagnosis.

Serum agglutination test is used for screening brucellosis and real-time PCR for the identification of *Brucella* DNA in serum samples (10). Complement fixation tests and RBT are used in combination to confirm brucellosis in many countries. The former test is used for its higher specificity, whereas the latter is used for its higher sensitivity. The primary binding assays include indirect fluorescent polarization assay (FPA), indirect enzyme-linked immunosorbent assay (iELISA), and the competitive ELISA (cELISA) that employ O-antigen or purified lipopolysaccharide (LPS) as the diagnostic reagent. Enzyme-linked immunosorbent assay tests have been developed to be more sensitive and specific alternatives to conventional tests (11). However, the gold standard in diagnosing brucellosis is culture (3). Data on risk factors for brucellosis among humans and animals are rare. However, risk factors associated with socio-demographic variables, animal contact practices, and dairy product consumption (milk and cheese) are attributed to human brucellosis. Regional traditional beliefs, as an indirect risk factor, may also affect the attitude toward the consumption of dairy products. These risk factors vary in different regions within and between countries, which contributes to the variation in the prevalence of brucellosis in varied geographical areas (12). Many occupations, such as butchers, ranchers/breeders, milkers, veterinarians, inoculators, and laboratory workers, as well as people involved in the packaging and sale of dairy products and raw meat, are at a high risk of brucellosis (10). Some people believe that treatments for brucellosis are very diverse and sufficient, but considering the high prevalence of this disease, it is a long way from eradication (13).

The prevalence of brucellosis is lower in developed countries than that in low-income countries (14). One of the most essential strategies to eradicate the disease is to use vaccinations. There is no human vaccine to prevent brucellosis because of multiple reasons, including infection after vaccination (15, 16). The best way to hinder the disease in humans is its prevention in animals (17). There is scant or no information on brucellosis in some developing countries (16). Veterinary complications and deaths from the infection cause substantial economic damage in these countries (4). In Kenya, Yemen, and Syria, the incidence of brucellosis is high. In recent years, the world's map of brucellosis has changed remarkably.

Even though several countries have made significant progress in controlling the disease, in some parts of the world, there is evidence of the emergence of new hotspots (18). *Brucella* has a special place in history, epidemiology, phylogenetics, and pathogenicity, which put it in the spotlight of scientists (19). This study aimed to evaluate the prevalence, diagnosis, and manifestations of brucellosis.

## Materials and methods

This study was performed according to the Preferred Reporting Items in Systematic Reviews and Meta-Analyses (PRISMA) guideline (Supplementary material 1). Using the keywords such as “*Brucella*,” “Brucellosis,” “Malta Fever,” “Bang disease,”' “*Brucella* Infection,” “'Mediterranean remittent fever,” “Undulant Fever,” and “Gibraltar Fever,” all relevant articles were collected (IR.MEDILAM.REC.1400.130).

### Method of literature search

A complete and comprehensive search was conducted in international databases, including PubMed, Web of Science, Scopus, Embase, and Google scholar, from 1 January 2001 to 5 August 2021. In the search, the following Medical Subject Headings (MeSH) keywords, including “Brucellosis,” “Malta Fever,” “Gibraltar Fever,” “Mediterranean fever,” “Cyprus Fever,” “Rock Fever,” “*Brucella* Infection,” and “Undulant Fever” were used. In the present study, the PICOS included population: people in all age groups, intervention: none, comparison: none, outcome: prevalence of brucellosis, time: 1 January 2001 until 5 August 2021, and study design: observational study. The search method explained below was used based on PICOS for MEDLINE (MeSH), followed by other databases:

1. *Brucella* [text word] OR *Brucella* [MeSH term]
2. Brucellosis [text word] OR Brucellosis [MeSH term]
3. Malta fever [text word] OR Malta Fever [MeSH term]
4. Undulant Fever [text word] OR Undulant Fever [MeSH term]
5. 1 OR 2 OR 3 OR 4
6. Prevalence [text word] OR Prevalence [MeSH term]
7. Frequency [text word] OR Frequency [MeSH term]
8. Incidence [text word] OR Incidence [MeSH term]
9. 6 OR 7 OR 8
10. 5 AND 9

By using Google Scholar, we accessed gray literature. In addition, we consulted a bacteriology expert to find related articles. In this regard, by hand searching, we found multiple articles from the references list of related articles. All extracted data were then imported into Endnote X6. Thereafter, the duplicated articles were removed and the remaining studies (original Articles/abstracts published in English) were screened. Afterward, the titles of the articles were examined, and if the article was relevant, its abstract and full text were reviewed. These steps were carried out by two independent raters, “R-P” and “S-KH,” and inter-rater differences were resolved by the opinion of “M-H.” The inter-rater reliability was 89%. Masking and task separation were strategies employed in the selection of the study procedure.

### Eligibility criteria

All cross-sectional, prospective, and retrospective case-series studies which reported the prevalence of brucellosis were included. However, case reports and case series with less than five patients (as the study population) and also clinical trial studies were excluded. Also, studies without reported prevalence data, definite sample sizes, and clear correct estimates of the prevalence, as well as case–control studies and abstracts presented in scientific meetings without full texts, were excluded from further study.

### Data extraction and quality assessment

In the current study, an excel data sheet was designed. From all eligible studies, general (author name, year, country, study design, sample size, or number of brucellosis patients, age, and sex) and specific (diagnosis method, therapy, symptoms, isolated bacteria, and comorbidities) data were extracted. The quality of these studies was appraised independently by two of the authors (R-P and S-KH) using the Joanna Briggs Institute checklist (20). The scale consisted of three parts, namely selection (four items), comparability (one item), and outcome (two items for cross-sectional and three items for cohort studies).

### Statistical analysis

All data used in this study were analyzed using Stata software 14.0 (College Station, Texas, USA). The number of brucellosis cases and sample size were considered the same as in previous studies (21, 22). Heterogeneity and heterogeneity quantification were determined with the aid of Cochran's *Q*-test and *I*^2^ index, respectively. Based on the Higgins classification approach, *I*^2^ values of more than 0.7 were regarded as high heterogeneity. The “Metaprop” command and random-effect model were applied to calculate the pooled prevalence with a 95% confidence interval (CI) and to estimate the pooled prevalence, respectively. The “Freeman–Tukey double-arcsine transformation” method was also utilized to estimate 95% CI to keep the values between 0 and 100%. Factors (age, sample size, gender, and year) affecting heterogeneity among studies were examined by the meta-regression analysis. The publication bias was also checked by the “metabias” command. In case of any publication bias, “meta-trim” command using the trim-and-fill method was used to adjust the prevalence rate. In all analyses, a level of 0.05 was considered statistically significant.

## Results

**Figure 1:**
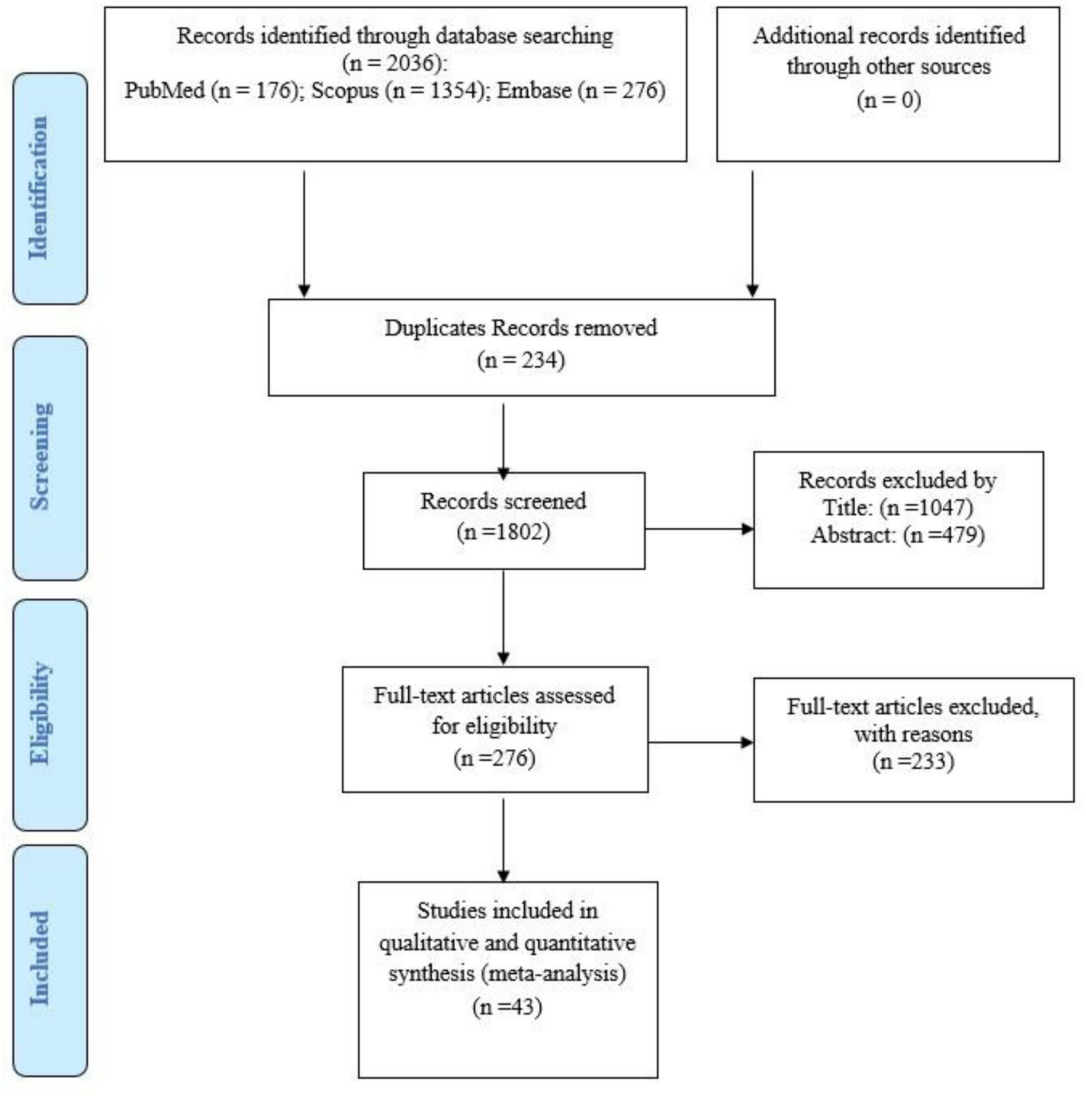
Flowchart of systematic review and meta-analysis.

Overall, 2,036 studies were found through databases. Following the exclusion of irrelevant articles, 1,802 studies were included. The screening was conducted in three steps. In the first step, 1,526 studies were excluded after reviewing the titles and abstracts. In the next step, the full texts of the remaining studies (*n* = 276) were evaluated and 233 studies were eliminated. In the last step, 43 studies with a total sample size of 52,502 were taken into consideration in the analysis. The flowchart of this selection process and the characteristic of studies are represented in Figure 1 and Table 1, respectively. Iran had the highest number of studies (*n* =18), followed by Pakistan with seven studies. The minimum age range of the subjects was 7.02 years in Pourakbari et al.'s (61) study, while the maximum age range was 68.7 years in the study of Sümer et al. (54). The results of the quality assessment of eligible studies are depicted in Supplementary Table 2. The quality of the studies was acceptable.

**Table 1:** Characteristics of studies included in this meta-analysis.

**References**	**Country**	**Enrollment time**	**Published time**	**Type of study**	**Number of patients**	**Gender**	**Mean age**	**Number of *Brucella*-positive patients**	**Gender**	**Diagnosis method**	**Signs and symptoms**	**Isolated bacteria**	**Prevalence %**
Ahmad et al. (23)	Pakistan	2013–2014	2014	Cohort	1,250	–	–	455	–	SAT, ELISA	–	*B. abortus*, *B.melitensis*	36.40
Esmaelili et al. (24)	Iran	2017–2019	2019	CS	289	M: 289	38	92	–	ELISA	–	–	31.84
Abdelbaset et al. (25)	Egypt	2017	2018	CS	53	M: 29 F: 24	26.5	5	–	RBPT, STAT	–	*B. abortus*, *B. melitensis*	9.434
Rezaee et al. (26)	Iran	2010–2011	2012	CS	1,541	M: 1,296 F: 238	37.5	222	M: 178 F: 44	SAT, CWT, AHGT, 2ME	Night sweats, orchitis back pain, headache, fever, myalgia, joints pain	–	14.41
Bamaiyi et al. (27)	Malaysia	2010–2011	2017	CS	446	–	40.62	6	M: 6	Coombs test	–	–	1.35
Beheshti et al. (28)	Iran	–	2010	CS	185	M: 185	36 (range: 11–70)	11	–	–	Fever, sweating, malaise, headache, low back pain, arthralgia, weakness restlessness	–	5.95
Alim et al. (29)	Turkey	2011	2015	CS	1,430	–	16–65	114	–	STAT, ELISA	Joint pain and distension, fever, night sweating, muscle and waist pain, weight lost, lymphadenopathy, hepatosplenomegaly, epididymis, orchitis	–	7.97
Khan et al. (30)	Pakistan	2014–2015	2017	CS	197	M: 148 F: 49	–	6		RBPT, ELISA	–	–	3.05
Khalili et al. (31)	Iran	2011	2012	CS	75		38.4 ± 6.4	44	–	ELISA	–	–	58.67
Ebrahimpour et al. (32)	Iran	2010	2012	CS	625		38.66	377	M: 205 F: 172	STAT, CWT, ELISA, 2ME	–	–	60.32
Rahman et al. (33)	Bangladesh	2007–2008	2012	CS	500	M: 375 F: 125	14–80	22	M: 21 F: 1	RBPT, STAT, ELISA, Real–time PCR	–	–	4.40
Ali et al. (34)	Pakistan	2011	2013	CS	262	M: 220 F: 42	33.3	18	M: 16 F: 2	RBPT, STAT	–	*B. abortus*	6.87
Sofian et al. (35)	Iran	2012	2013	CS	163	M: 81 F: 82	25	15	M: 7 F: 8	STA, 2ME	–	–	9.20
Arvas et al. (36)	Turkey	2010	2013	CS	2,913	M: 889 F: 2,024	≥18	525	M: 145 F: 380	STA, WAT	–	–	18.02
Shakurnia et al. (37)	Iran	2011	2014	CS	1,450	M: 1,405 F: 45	37 ± 9.5	5	–	RBPT, SAT, 2ME	–	–	0.345
Workalemahu et al. (38)	Ethiopia	2014	2017	CS	254	M:200 F: 43	26 ± 8.26	27	–	STAT	–	–	10.63
Ntirandekura et al. (39)	Tanzania	2017–2018	2020	CS	28	–	–	9	–	Real–time PCR, RBPT, ELISA, FPA	–	*B. melitensis*, *B. abortus*, *B. suis*	32.15
Ali et al. (40)	Pakistan	2016	2018	CS	250	M: 125	10–70	40	–	RBPT, IgM—ELISA	–	–	16.00
Alshehabat et al. (41)	Jordan	–	2019	CS	185	M: 139 F: 46	–	0	–	D-Tec^®^ CB RSAT kit with secondary 2ME	–	*B. canis*	0.0
Yousaf et al. (42)	Pakistan	2014–2015	2021	CS	218	M: 82 F: 1,366	–	37	–	RBPT, Real-time PCR	Fever, severe back and joint pain, headache, weakness, loss of appetite, depression	–	16.97
Migisha et al. (43)	Uganda	2017	2018	CS	235	–	–	45	–	RBPT, Blood culture	Headache, joint or back pains and chills, hepatomegaly, splenomegaly, low BMI	–	19.15
Mohseni et al. (44)	Iran	2015	2017	CS	100	–	–	50	M: 30 F: 20	SAT, WAT, ELISA	Headache, fever, chills, fatigue, joint pain, back pain	–	50.00
Salmanzadeh et al. (45)	Iran	2020	2021	CS	100	–	40.12	17	M:100	ELISA, IBL kit tubular agglutination method	–	–	17.00
Paronyan et al. (46)	Armenia	2010–2012	2016	CS	600	–	35.5	50	M: 41 F: 8	Wright, Huddleston, RBPT, PCR, ELISA	Fever, fatigue, diarrhea, nausea/vomiting, shaking/rigors, skin lesions, pain behind the eyes, unusual bleeding, neck stiffness, abdominal tenderness, pharyngeal, lymphadenopathy, jaundice, mental status change, joint effusions, conjunctival infection, neurological findings, bleeding, neck stiffness, heart murmur	–	8.34
Parizadeh et al. (47)	Iran	2005–2006	2009	CS	908	M: 789 F: 118	–	275	–	SAT, CWT, 2ME	–	–	30.29
Saddique et al. (48)	Pakistan	2014–2015	2019	CS	446	M: 230 F: 216	36.55	45	M: 17 F: 28	RBPT, Real–time PCR	–	*B. abortus*	10.09
Akbarian et al. (49)	Afghanistan	2012– 2013	2015	CS	1,017	–	–	53	–	RBPT, ELISA	–	–	5.21
Honarvar et al. (50)	Iran	2015	2017	CS	536	M: 220 F: 316	35.5	54	M: 19 F: 35	SAT, 2ME, CWT	Fever, chills, night sweats, headache, low back pain, arthralgia, myalgia	–	10.08
Mukhtar et al. (51)	Pakistan	–	2010	CS	360	–	34.36	78	M: 78	ELISA	–	–	21.67
Mendoza-Núñez et al. (52)	Peru	2005–2006	2008	CS	206	–	–	14	–	RBT, *Brucella* IgM/IgG flow assay, STAT, Blood culture	Skeletal complaint, skeletal involvement, hepatic, articular, dermatologic, hematologic, genitourinary involvement	*B. melitensis*	6.80
Mangalgi et al. (53)	India	2008–2012	2016	CS	2,337	–	–	222	–	RBPT, SAT, 2ME	Fever, joint pain, low backache, myalgia, night sweating, fatigue, headache, weight loss, orchitis	–	9.50
Sümer et al. (54)	Turkey	2002	2003	CS	750	M: 368 F: 382	68.7	24	M: 12 F: 12	WAT	–	*B. melitensis*	3.20
Zadsar et al. (55)	Iran	–	2019	CS	14,706	M: 13,571 F: 1,135	33.5	11	–	STA, 2ME	–	–	0.08
Mantur et al. (56)	India	1988–2001	2004	CS	5,726	–	10.12	93	M: 73 F: 20	STAT	Fever, joint pain, low backache, jerky movements of limbs, burning feet, splenomegaly, hepatomegaly, hepatosplenomegaly, pityriasis alba, skin lesions, carditis, chorea, meningitis, peripheral neuritis.	*B. melitensis* biotype 1 and 3, *B.agglutinins*	1.63
Dutta et al. (57)	India	2013–2015	2017	CS	236	–	13.72	29	M: 15 F: 14	STAT, RBPT, IgM ELISA, IgG ELISA, PCR	Joint pain, low back pain, fatigue and night sweat, fever	–	12.29
Keramat et al. (58)	Iran	2016	2019	Cohort	2,367	M: 1,060 F: 1,303	34.57	238	–	Wright, 2ME	–	–	10.05
Hajia et al. (59)	Iran	2009–2010	2013	Cohort	267	–	37.05	110	M: 64 F: 46	SAT, CWT, 2ME, ELISA, PCR	–	*B. abortus, B. melitensis*	41.20
Kazemi et al. (60)	Iran	–	2008	CS	104	M: 43 F: 61	34.97	84	M: 40 F: 44	RBT, WAT, Culture, PCR	–	*B. melitensis* biovar Suis	80.77
Pourakbari et al. (61)	Iran	2011 and 2016	2019	Cohort	8,018	–	7.02	43	M: 26 F: 17	SAT, Wright test, CWT, 2ME, culture, CT, MRI, Gram staining, biochemical tests	Fever, weight loss, anorexia, malaise, fatigue, arthralgia, sweating, nausea and vomiting, cough, pain, headache, diarrhea, splenomegaly, hepatomegaly, insomnia, amenorrhea, incontinence, abdominal neck stiffness, confusion, depression	–	0.54
Etemadi et al. (62)	Iran	2017–2018	2020	CS	297	M:164 F:133	41.4	141	M: 77 F: 64	Multiplex PCR, RBT, standard tube, blood culture and phage typing agglutination test	Fever, sweats, headache, arthralgia, backache and myalgia	*B. melitensis* biovar 1	47.48
Sabour et al. (63)	Iran	2018–2019	2020	CS	173	–	34.8	60	M: 33 F: 27 F	Brucellacapt test, real-time PCR, RBT, STA, 2ME	Fever, fatigue, weight loss, profuse sweating, anorexia, headache, arthralgia, and generalized aching	–	34.68
Guzmán-Bracho et al. (64)	Mexico	2014–2016	2020	CS	558	–	–	222	–	RBT, SAT, 2ME, ELISA, LFT, brucellacapt	Fever, headache, arthralgia	–	39.78
Sanodze et al. (65)	Georgia	2009–2011	2015	CS	141	–	35	27	M: 25 F: 2 F	SAT, Real-time PCR	Fever, sweats, rigors, malaise, fatigue, anorexia, weight loss, arthralgia, myalgia, arthritis, neuritis, neuro-psychiatric symptoms, epididymo-orchitis, changes in LFT	*B. melitensis, B. abortus*	19.15

CS, cross-sectional; F, female; M, male; SAT, serum agglutination test; WAT, Wright's agglutination test; STA, standard tube agglutination; CWT, Coombs Wright test; RBT, Rose Bengal test; ELISA, enzyme-linked immunosorbent assay; RBPT, Rose Bengal plate test; STAT, serum tube agglutination test; 2ME, 2-mercaptoethanol; AHGT, anti-human globulin (Coombs) test; FPA, fluorescence polarization assay; LFT, liver function test; PCR, polymerase chain reaction.

### Pooled prevalence of brucellosis

**Figure 2:**
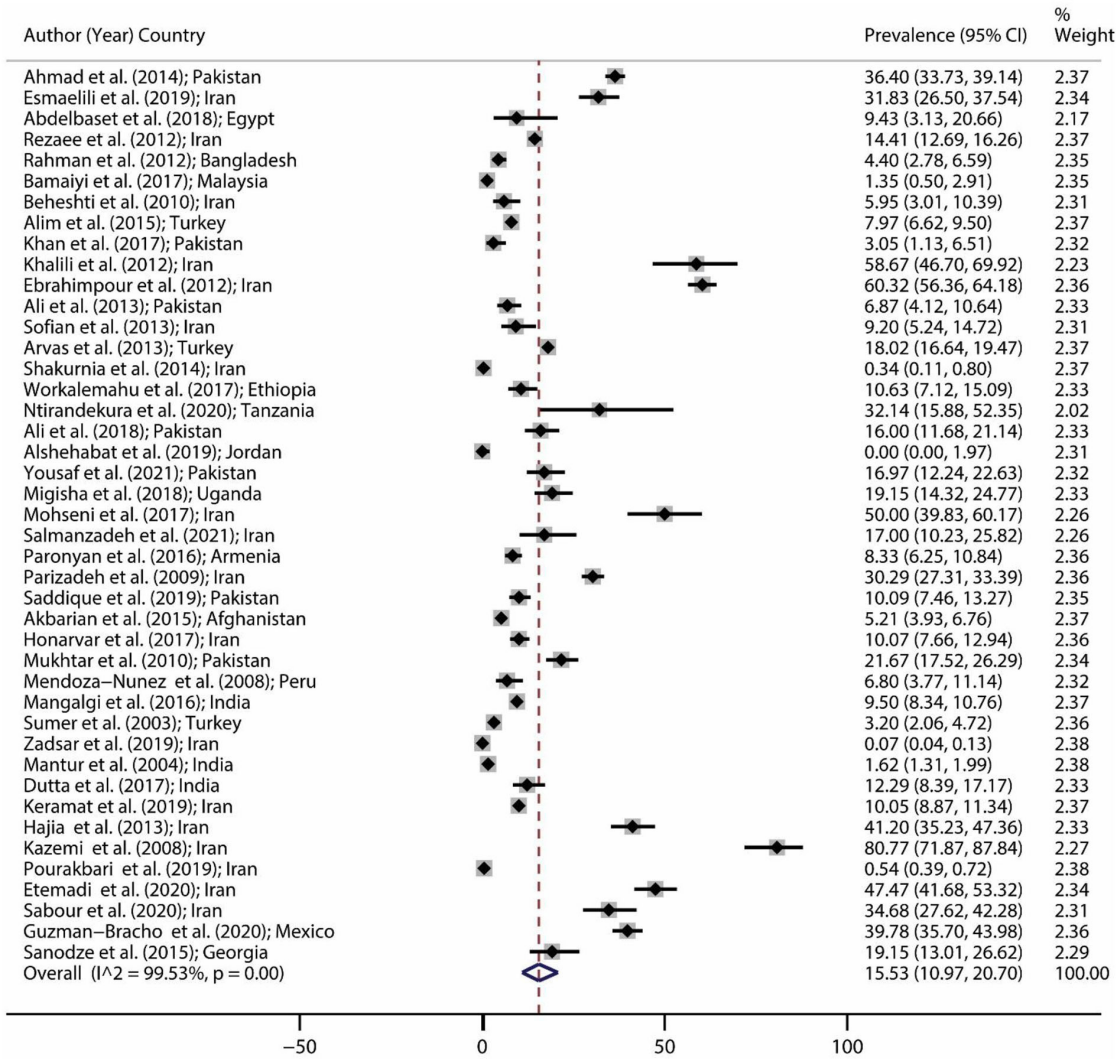
Forest plot for brucellosis prevalence in the world based on a random-effect model. Each study identifies the first author (year) and country. Each line segment's midpoint shows the prevalence estimate, the length of the line segment indicates a 95% confidence interval (CI) in each study, and the diamond mark illustrates the pooled estimate.

The brucellosis prevalence in all eligible studies and the forest plot of brucellosis prevalence are illustrated in Table 1 and Figure 2, respectively. The minimum prevalence (0.0%; 95% CI: 0–1.97) of brucellosis was reported by Alshehabat et al. (41) in Jordan, while the maximum prevalence (80.77%; 95% CI: 71.87–87.84) was reported by Kazemi et al. (60) in Iran. Based on the results of the random-effect model approach, as shown in Figure 2, the pooled estimate for brucellosis prevalence was 15.53% (95% CI: 10.97–20.70).

### Pooled prevalence of brucellosis based on gender and country

**Figure 3:**
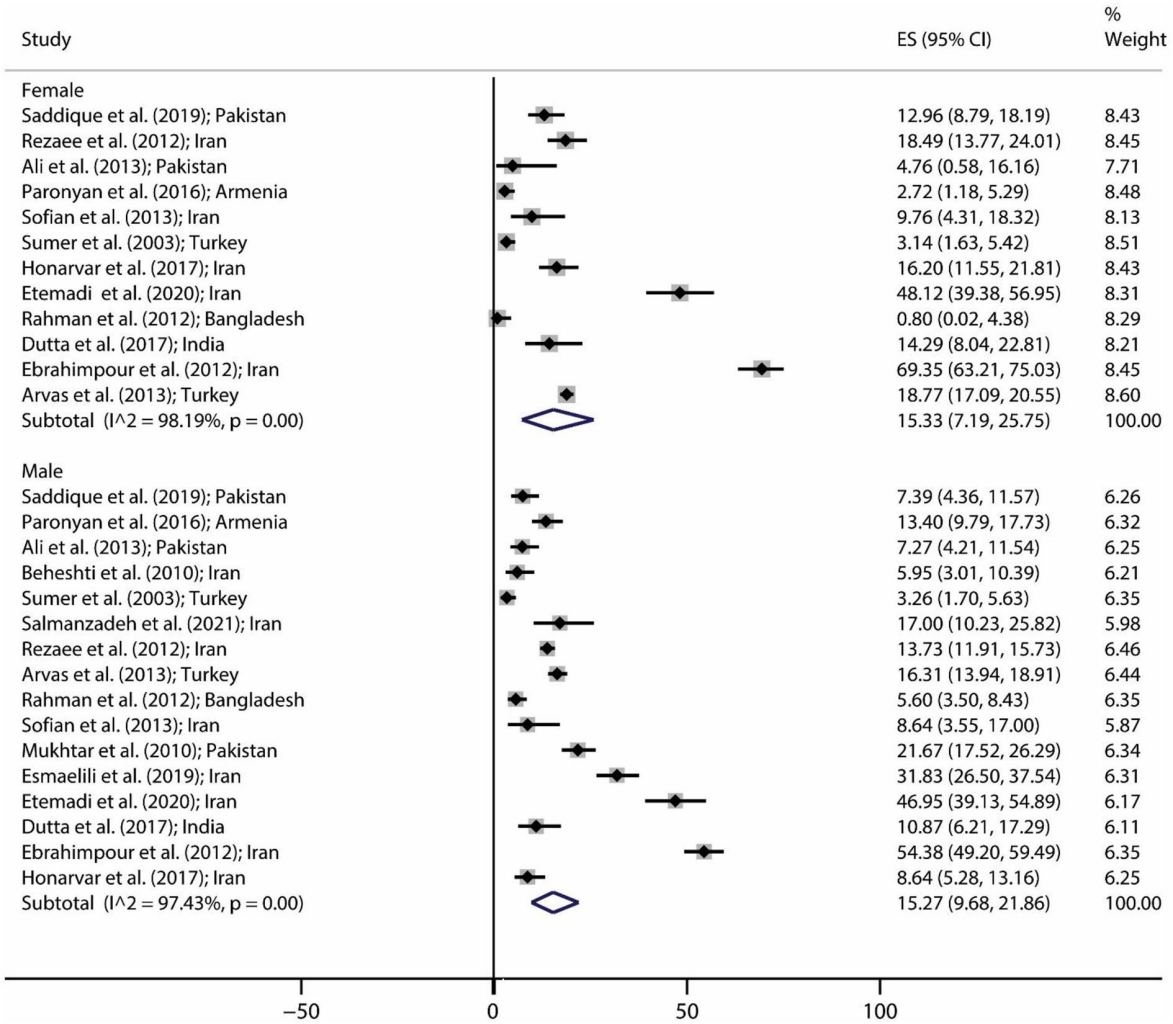
Pooled prevalence with 95% confidence interval (CI) of brucellosis prevalence based on gender. The diamond mark shows the pooled prevalence, and the length of the diamond indicates the 95% CI.

**Figure 4:**
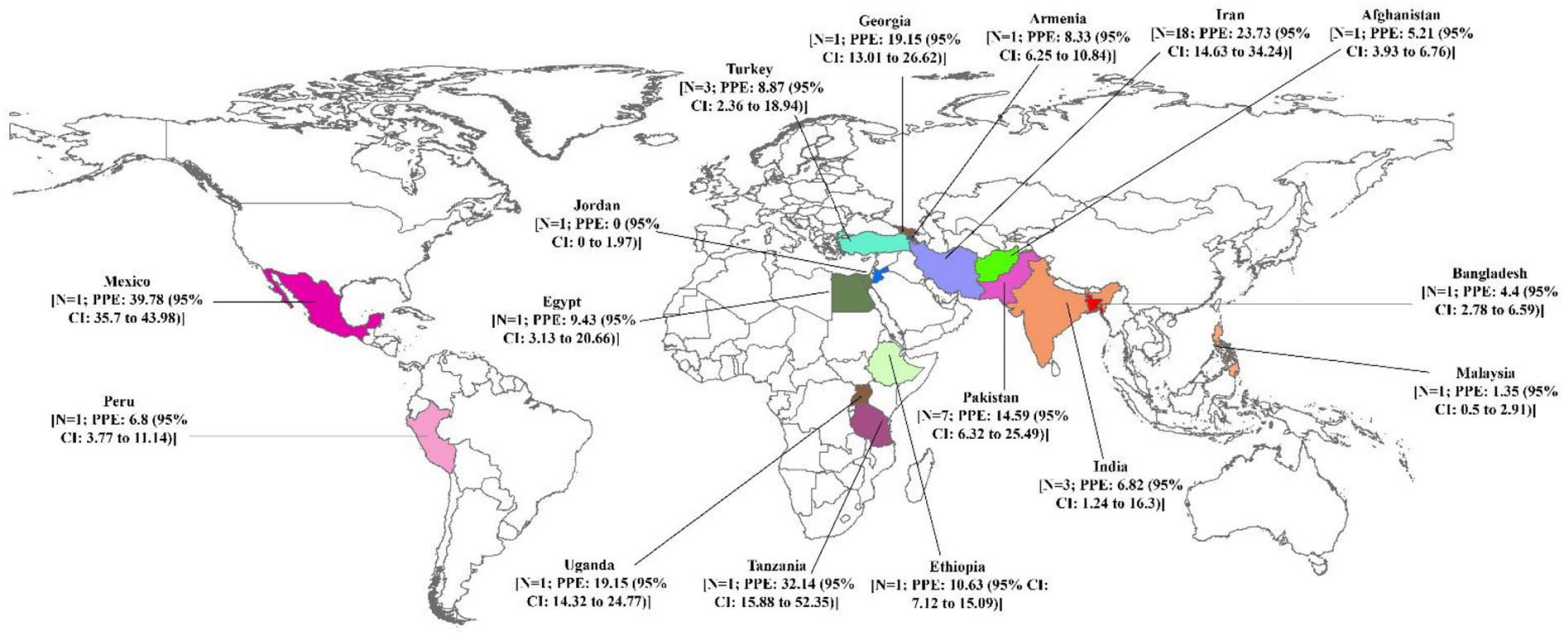
Pooled prevalence with 95% confidence interval (CI) of brucellosis prevalence based on country.

The pooled prevalence of brucellosis based on gender is demonstrated in Figure 3. The number of investigations on men and women was 16 and 12 studies, respectively. The pooled prevalence of brucellosis was 15.27% (95% CI: 9.68–21.86; heterogeneity *I*^2^ index: 97.43; *p* < 0.001) for men and 15.33% (95% CI: 7.19–25.75; heterogeneity *I*^2^ index: 98.19; *p* < 0.001) for women. Figure 4 shows the pooled prevalence of brucellosis based on country. As mentioned earlier, Iran has the highest number of studies (*n* = 18). The highest and lowest prevalence of brucellosis was observed in Mexico (39.78%; 95% CI: 35.7–43.98) and Jordan (0%; 95% CI: 0–1.97), respectively.

### Heterogeneity and meta-regression

**Figure 5:**
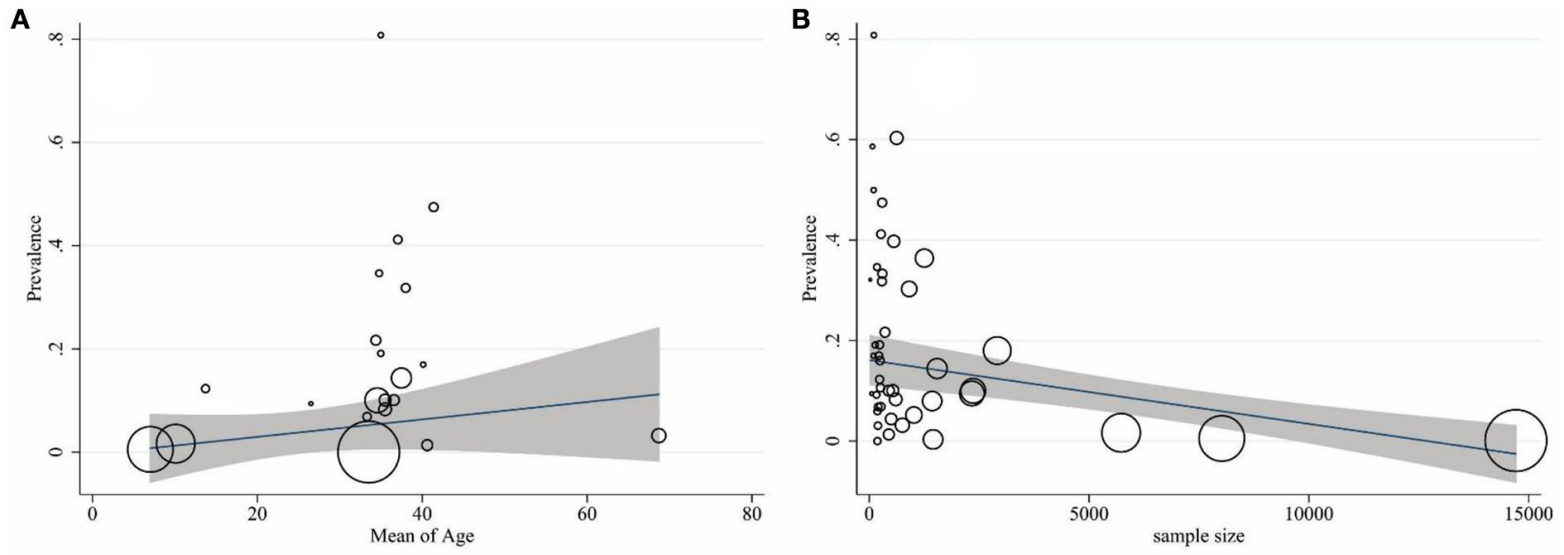
Association of the prevalence of age **(A)** and sample size **(B)** with that of brucellosis by using meta-regression. The size of the circles denotes the precision of each study.

The results of heterogeneity are displayed in Table 2. Cochran's *Q*-test indicated significant heterogeneity among studies (*p* < 0.001). It means that the reported prevalence varies in primary studies and the main source of variation is caused by the difference in the true effects. The *I*^2^ index for the total prevalence of brucellosis was 99%. In other words, more than 99% of the variance in this study was due to heterogeneity. The results of the meta-regression exhibited that the sample size (coefficient: −0.002; *p* = 0.044) significantly affected heterogeneity among studies. The age (coefficient: 0.240; *p* = 0.480), gender (coefficient: −0.017; *p* = 0.800), publication year (coefficient: 0.114; *p* = 0.861), and quality of studies (coefficient: 0.034; *p* = 0.117) showed no significant effect on heterogeneity (Figures 5A, B).

**Table 2:** The univariate meta-regression analysis on the heterogeneity of the determinants in included studies for the prevalence of brucellosis.

**Variables**	**Coefficient**	**95% CI**	***p*-value**
Age	0.240	−0.457 to 0.938	0.480
Sample size	−0.002	−0.004 to −0.001	0.044^*^
Gender	−0.017	−0.149 to 0.117	0.800
Publication year	0.114	−1.418 to 1.191	0.861
Quality score	0.034	−0.009 to 0.077	0.117

CI, Confidence interval.

* Significance at level of 0.05.

### Publication bias and trim-and-fill method

**Figure 6:**
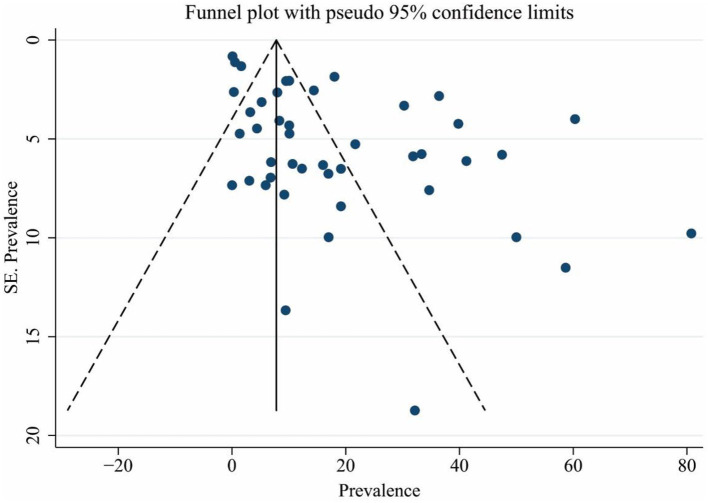
Funnel plot for assessing publication bias. The vertical and horizontal axes show the prevalence of brucellosis and the standard error of prevalence.

Based on the results of Egger's test, a significant publication bias was observed for the prevalence of brucellosis (coefficient: 3.894; *p* < 0.001). The funnel plot (Figure 6) showed that some evidence might be missed due to not getting published. Therefore, the trim-and-fill-adjusted prevalence of brucellosis (18.30%; 95% CI: 14.10–22.52) was generated, which did not significantly differ from the original prevalence of brucellosis (15.53%; 95% CI: 10.97–20.70).

## Discussion

While brucellosis and its transmission routes have been discovered for more than 100 years, it is still a global concern, especially in low-income countries (1). Human brucellosis happens through direct or indirect contact with infected animals or their products. Various symptoms, such as fever, osteoarthritis, fatigue, and sweating, may occur in patients (66). Fever, back pain, cough, gastrointestinal symptoms, and blood disorders are multiple manifestations of brucellosis similar to those of other diseases, which often lead to misdiagnosis (3). For the treatment of brucellosis, a combination of tetracycline with rifampicin for 6 weeks or an alternative treatment with fluoroquinolone or co-trimoxazole in combination with rifampicin is commonly used. Medication reduces the length of the disease and prevents recurrence and complications such as arthritis and endocarditis (67). Given the importance and prominent position of brucellosis in the economies of countries, in this study, we evaluated the prevalence of brucellosis and its epidemiology. Transmission and prevalence of brucellosis depend on various factors comprising eating habits, processing methods of milk and its products, social customs, animal husbandry methods, climatic conditions, social and economic status, and environmental health (68).

The highest prevalence of brucellosis has been reported in countries, such as West Asia, India, the Middle East, Southern Europe, and Latin America. The main cause of the disease is *B. melitensis* (1), which has been eradicated in countries, such as Australia, Canada, Israel, Japan, and New Zealand (2). In industrialized and developed countries, the prevalence of brucellosis is low due to the careful screening program of infected animals and the vaccination of livestock (1). A number of countries have also attempted to eradicate brucellosis by implementing the following measures: setting up a strong system for continuous monitoring of animals and recording positive cases, close monitoring of slaughterhouses, markets, herds for quick and timely identification of positive cases, strict monitoring of herd movement to prevent the spread of infection, continuous and formal training of veterinarians, farmers, and supervisors, compensation of farmers in case of destruction of animals, and establishment of laws to support eradication programs to inhibit non-compliance with official actions (69). For four decades, developed countries have eradicated the infection using control and eradication programs, such as compulsory livestock vaccination; however, following this attempt, they limited and finally banned vaccination. In the continuation of the eradication program in developed countries, the test-and-slaughter policy was implemented, which usually requires more than 10 years to be completed, but the important point in the success of this plan is to compensate the financial losses of farmers (16). The Eastern Mediterranean, including countries, such as Iran, Jordan, Egypt, Palestine, Syria, and Lebanon, is an endemic area of brucellosis. More than half a million people in these countries are infected with brucellosis annually (70).

In endemic areas, there are several reasons for the persistence of infection, including low vaccination, differences between the number of positive animals expected and sampled due to insufficient monitoring, negative herds becoming positive due to the lack of continuous control, refusal of farmers to remove positive livestock owing to non-payment of compensation by regulatory agencies, cessation of eradication projects because of various reasons, for example, the absence of sufficient funds, recurrence of infection due to the lack of permanent monitoring, and problems in detecting positive cases arising from the low prevalence of infection (71). In developing countries, innovative approaches are needed; otherwise, it will be impossible to implement an effective eradication policy. Mongolia, with mass vaccination of livestock, and Tajikistan, with biennial vaccination of small ruminants with Rev1, reduced the prevalence of infection from 25.1 to 7.5% (16). In addition to the reasons mentioned earlier for the non-eradication of brucellosis, there are other reasons, such as the lack of public awareness of Malt fever, the absence of a proper eradication program, limited availability of animal vaccines in the market, the absence of a human vaccine, the maintenance of vaccinated animals more than the vaccine protection period, the use of traditional animal husbandry system, proximity of animal shelters to living areas, lack of border quarantine for the entry of livestock, and living in developing countries, which cause infection among animals and subsequently humans (72). The Middle East is a region with a high incidence of brucellosis, and its native countries, such as Syria, Iraq, Saudi Arabia, Turkey, and Iran, have the most reports of the disease (73).

In the present study, the highest (80.76%) and lowest (0%) prevalence of brucellosis was related to Iran and Jordan, respectively. The reason for the high incidence of the infection is that the majority of eligible articles have been published in Iran. According to the meta-analysis conducted in this study, the highest and lowest prevalence by country and the number of studies belonged to Mexico (39.78%) and Jordan (0%), respectively. The majority of countries surveyed in this study had only one article. Of note, if further studies are performed in a country, and if investigations are conducted in additional countries and different regions of the world, more accurate statistics on the prevalence of brucellosis can be provided. Unfortunately, in many countries that are economically poor and, of course, financial poverty affects the incidence of brucellosis, less information on brucellosis prevalence is available. The low incidence of the disease in some endemic areas is due to the lack or low level of monitoring and reporting (1); there is no exact estimate of the annual incidence of human brucellosis. The international community should allocate resources to understand and fill gaps in information because the existence of this information helps determine effective control strategies. The World Health Organization (WHO) will greatly contribute to the progress of this process by restoring human brucellosis as a neglected zoonotic disease priority (74).

According to the WHO, the prevalence of infection in the world varies between 0.01 and 200 people per 100,000 people. In the United Kingdom, the prevalence is 0.3 per million and in Germany, the prevalence is 0.03 per 100,000 (70). In this study, the global prevalence of brucellosis was estimated at 15.53%. Previous studies have shown that the prevalence of brucellosis is higher in men than in women. Occupational exposure and gender differences in access to healthcare have been cited as factors influencing this variation (1, 14). In the present study, gender had no effect on heterogeneity, and no significant difference was observed between the two gender groups. However, the prevalence was slightly higher in women (15.33%) than in men (15.27%).

In this systematic review, we found that the average age range varies from 7.02 to 68.7. In a review conducted in Kyrgyzstan, the results of a 10-year analysis revealed that all age groups, most of which were active workers, were affected by brucellosis (75). Brucellosis is detected in all age groups but is usually rare in children (1). Pediatric brucellosis is uncommon in areas where *B. abortus* is endemic, but in endemic areas of *B. melitensis*, cases are seen in children. One of the most important causes of infection in children is the consumption of raw milk (56). Socio-economic factors, improvement of monitoring systems, and animal control programs have led to the evolution of the epidemiology of human brucellosis (14). Certain population groups of countries, including immigrants and people with poor socio-economic status, are frequently more exposed to brucellosis than others (70). Another factor influencing the epidemiology of brucellosis is political change. For instance, before the collapse of the Soviet Union in 1991, the country was able to control the disease to a large extent, but after the dissolution of the Soviet Union, the prevalence increased in independent countries (14).

The incidence and prevalence of brucellosis can vary not only in different countries but also within a country (14). In a study conducted in Iran, the incidence of brucellosis was reported to be between 7.0 and 276.41 cases per 100,000 people (70). Various studies have also reported that the incidence of brucellosis depends on the seasons. Studies in Iran, Turkey, and Germany showed that the highest prevalence of the disease was in spring and summer, and the least occurred in winter and autumn (76–79). Spring and summer are the calving and lactation seasons for sheep (75, 80). In addition, during these seasons, the production and consumption of milk and dairy products increase, which, in turn, elevates the risk of brucellosis (80). To prevent the spread of the disease, a series of preventive measures should be taken into consideration. Since brucellosis is a zoonotic disease, it is critically important to control the disease in the animal host. Brucellosis is transmitted through the consumption of food, especially contaminated dairy products, which indicates the importance of heat treatment of dairy products. Another important point is occupational dependency in disease transmission, highlighting the need for appropriate and adequate health measures in people related to animals (1). For many years, two successful vaccines (Rev1 and *B. abortus* S19) have been used for the control of brucellosis in the world, but there are still disadvantages, such as abortion, bacterial removal from vaccinated animals, and induction of disease in humans (81). Appropriate control and preventive measures cause a significant decrease in the incidence of brucellosis. In a report published from Kyrgyzstan, the number of human brucellosis cases and its incidence increased between the years 1994 and 2010, but between 2010 and 2020, there was a continuous decline, which is due to effective preventive and control measures (75).

Limitations can be placed on this meta-analysis and systematic review. In this study, we observed the lack of high-quality scientific data and the absence of sufficient information from many countries, which did not allow a comprehensive analysis of all regions of the world. Another limitation was that we included only articles published in English. This is an important issue because, in many indigenous areas of brucellosis, articles have been published in the particular language of the region. Therefore, these articles have not been considered and evaluated in the present study. Despite many studies on brucellosis, the disease remains a health, economic, and social problem in many parts of the world, particularly in developing and low-income countries. Careful screening of brucellosis and its prevalence in the world and in different countries lead to accurate monitoring of the disease and its epidemic. Brucellosis is a long way from eradication. The important thing in controlling the disease is the strategies used for its prevention. Vaccination is the main strategy to control the disease (71). Prevalence rate, type of animal husbandry, availability of the vaccine, quality of consumed vaccine, amount of available financial and human resources and also legal authority, intersectoral cooperation, and surveillance identification are influential in the successful control of brucellosis (82).

While there is no human vaccine for brucellosis, regular and accurate vaccination of cattle can prevent this zoonotic disease. Educating people, especially villagers, about how to properly sterilize milk and its products can be a promising way to prevent the spread of the disease. With widespread alterations that occur annually in different parts of the world, the prevalence of brucellosis is changing and must be constantly monitored. An effective step to control brucellosis in future is to design and construct a novel vaccine that can create proper immunity in humans. Therefore, to encourage researchers to do this, an international call for the development of a new brucellosis vaccine with a significant price should be considered. In this call, researchers and scientists must compete to create the first licensed human vaccine (81).

## Limitation

Some effective factors in the prevalence of brucellosis were not covered in our study due to the lack of data, which is considered a significant limitation. Heterogeneity between the studies is another limitation. Therefore, we applied a random-effect model to combine the primary results in this meta-analysis. Studies written in English and the lack of studies from some regions of the world are other limitations of the present study.

## Conclusion

The epidemiology of brucellosis is important because it is a common disease between humans and animals and can be transmitted to humans in different ways. In this study, the prevalence of brucellosis and factors affecting its incidence were investigated. The prevalence of brucellosis is closely related to the economic, cultural, and even educational status of communities. In developed countries, unlike developing ones, brucellosis is lower. Given this issue, the need for more extensive studies in low-income countries and the pursuit of strict control and prevention programs are recommended. A very prominent point that can be taken into account from the results of this study is that there are no data from some countries due to the lack of sufficient publication, which causes a big gap in the estimation of the annual prevalence of brucellosis in the world. To solve this problem, researchers need to cooperate in conducting more studies on brucellosis and subsequently publishing their data. The most significant matter about the eradication and prevention of brucellosis is the cooperation of the government and international sectors because this disease can affect the economy of countries so much that if these organizations do not take effective actions, many problems will befall the deprived and poor people.

## Data availability statement

The original contributions presented in the study are included in the article/Supplementary material, further inquiries can be directed to the corresponding author.

## Author contributions

SK, RP, MK, MS, AA, GI, MM, IP, NS, and MH contributed to the revisions of the article. All authors approved the final version of the manuscript for publication.
