# Supplementary material for: Prevalence, diagnosis, and manifestations of brucellosis: A systematic review and meta-analysis
Source: Front Vet Sci. 2022 Dec 22;9:976215. doi: 10.3389/fvets.2022.976215 (PMC9813401; doi:10.3389/fvets.2022.976215)
Supplement: Supplementary file 2 [file Table_2.docx]

***Supplement table 2. Quality assessment for validation studies on the* global prevalence of brucellosis*.***

| **First author** | **Year** | Was the sample representative of the target population? | Were study participants recruited in an appropriate way? | Was the sample size adequate? | Were the study subjects and the setting described in detail? | Was the data analysis conducted with sufficient coverage of the identified sample? | Were objective, standard criteria used for the measurement of the condition? | Was the condition measured reliably? | Are all important confounding factors/subgroups/differences identified and accounted for? | Were subpopulations identified using objective criteria? | Was there appropriate statistical analysis? | QS |
| --- | --- | --- | --- | --- | --- | --- | --- | --- | --- | --- | --- | --- |
| Ahmad et al. | 2014 | Yes | No | No | Yes | Yes | Yes | Yes | No | No | Yes | 6 |
| Esmaelili et al. | 2019 | Yes | Yes | Yes | Yes | Yes | Yes | Yes | Yes | Yes | Yes | 10 |
| Abdelbaset et al. | 2018 | Yes | No | Yes | Yes | Yes | Yes | Yes | Yes | Yes | Yes | 9 |
| Rezaee et al. | 2012 | No | No | Yes | Yes | Yes | Yes | Yes | No | Yes | Yes | 7 |
| Bamaiyi et al. | 2017 | No | No | Yes | Yes | Yes | Yes | Yes | No | Yes | Yes | 7 |
| Beheshti et al. | 2010 | Yes | Yes | Yes | No | Yes | Yes | Yes | No | No | Yes | 7 |
| Alim et al. | 2015 | Yes | Yes | Yes | No | Yes | Yes | Yes | No | No | Yes | 7 |
| Khan et al. | 2017 | Yes | Yes | Yes | Yes | Yes | Yes | Yes | No | No | Yes | 8 |
| Khalili et al. | 2012 | Yes | Yes | Yes | Yes | Yes | Yes | Yes | Yes | Yes | Yes | 10 |
| Ebrahimpour et al. | 2012 | Yes | No | No | Yes | Yes | Yes | Yes | No | No | Yes | 6 |
| Rahman et al. | 2012 | Yes | Yes | Yes | Yes | Yes | Yes | Yes | No | No | Yes | 8 |
| Ali et al. | 2013 | Yes | No | Yes | No | Yes | Yes | Yes | No | No | Yes | 6 |
| Sofian et al. | 2013 | Yes | No | Yes | No | Yes | Yes | Yes | No | No | Yes | 6 |
| Arvas et al. | 2013 | Yes | No | No | Yes | Yes | Yes | Yes | No | No | Yes | 6 |
| Shakurnia et al. | 2014 | No | Yes | Yes | Yes | Yes | Yes | Yes | No | No | Yes | 7 |
| Workalemahu et al. | 2017 | Yes | Yes | Yes | No | Yes | Yes | Yes | No | No | Yes | 7 |
| Ntirandekura et al. | 2020 | Yes | Yes | Yes | Yes | Yes | Yes | Yes | No | Yes | Yes | 9 |
| Ali et al. | 2018 | Yes | Yes | Yes | Yes | Yes | Yes | Yes | Yes | Yes | Yes | 10 |
| Alshehabat et al | 2019 | No | No | Yes | Yes | Yes | Yes | Yes | No | No | Yes | 6 |
| Yousaf et al. | 2021 | Yes | Yes | Yes | Yes | Yes | Yes | Yes | No | No | Yes | 8 |
| Migisha et al. | 2018 | No | No | No | Yes | Yes | Yes | Yes | Yes | Yes | Yes | 7 |
| Mohseni et al. | 2017 | Yes | Yes | Yes | No | Yes | Yes | Yes | No | No | Yes | 7 |
| Salmanzadeh et al. | 2021 | Yes | Yes | Yes | Yes | Yes | Yes | Yes | No | No | Yes | 8 |
| Paronyan et al. | 2016 | Yes | No | No | Yes | Yes | Yes | Yes | Yes | Yes | Yes | 8 |
| Parizadeh et al. | 2009 | Yes | No | Yes | No | Yes | Yes | Yes | No | No | Yes | 6 |
| Saddique et al. | 2019 | Yes | Yes | No | Yes | Yes | Yes | Yes | Yes | Yes | Yes | 9 |
| Akbarian et al. | 2015 | Yes | Yes | Yes | Yes | Yes | Yes | Yes | No | No | Yes | 8 |
| Honarvar et al. | 2017 | Yes | Yes | Yes | Yes | Yes | Yes | Yes | Yes | Yes | Yes | 10 |
| Mukhtar et al. | 2010 | Yes | No | No | Yes | Yes | Yes | Yes | No | No | Yes | 6 |
| Mendoza-Núñez et al. | 2008 | Yes | Yes | Yes | Yes | Yes | Yes | Yes | No | No | Yes | 8 |
| Mangalgi et al. | 2016 | Yes | No | Yes | No | Yes | Yes | Yes | No | No | Yes | 6 |
| Sümer et al. | 2003 | Yes | No | Yes | No | Yes | Yes | Yes | No | No | Yes | 6 |
| Zadsar et al. | 2019 | Yes | No | No | Yes | Yes | Yes | Yes | No | No | Yes | 6 |
| Mantur et al. | 2004 | No | Yes | Yes | Yes | Yes | Yes | Yes | No | No | Yes | 7 |
| Dutta et al. | 2017 | Yes | Yes | Yes | No | Yes | Yes | Yes | No | No | Yes | 7 |
| Keramat et al. | 2019 | Yes | Yes | Yes | Yes | Yes | Yes | Yes | No | Yes | Yes | 9 |
| Hajia et al. | 2013 | 2017 | Yes | Yes | Yes | No | Yes | Yes | Yes | No | No | 7 |
| Kazemi et al. | 2008 | 2020 | Yes | Yes | Yes | Yes | Yes | Yes | Yes | No | Yes | 9 |
| Pourakbari et al. | 2019 | 2018 | Yes | Yes | Yes | Yes | Yes | Yes | Yes | Yes | Yes | 10 |
| Etemadi | 2020 | 2019 | No | No | Yes | Yes | Yes | Yes | Yes | No | No | 6 |
| Sabour | 2020 | 2021 | Yes | Yes | Yes | Yes | Yes | Yes | Yes | No | No | 8 |
| Guzmán-Bracho | 2020 | 2018 | No | No | No | Yes | Yes | Yes | Yes | Yes | Yes | 7 |
| Sanodze | 2015 | 2017 | Yes | Yes | Yes | No | Yes | Yes | Yes | No | No | 7 |
